# The Passage of H_2_O_2_ from Chloroplasts to Their Associated Nucleus during Retrograde Signalling: Reflections on the Role of the Nuclear Envelope

**DOI:** 10.3390/plants11040552

**Published:** 2022-02-19

**Authors:** Emily Breeze, Philip M. Mullineaux

**Affiliations:** 1 School of Life Sciences, University of Warwick, Coventry CV4 7AL, UK; emily.breeze@warwick.ac.uk; 2 School of Life Sciences, University of Essex, Wivenhoe Park, Colchester, Essex CO4 3SQ, UK

**Keywords:** retrograde signalling, chloroplasts, nucleus, endoplasmic reticulum, hydrogen peroxide, nuclear envelope, peri-nuclear space, aquaporins, membrane contact sites, cytoskeleton, environmental stress

## Abstract

The response of chloroplasts to adverse environmental cues, principally increases in light intensity, stimulates chloroplast-to-nucleus retrograde signalling, which leads to the induction of immediate protective responses and longer-term acclimation. Hydrogen peroxide (H_2_O_2_), generated during photosynthesis, is proposed to both initiate and transduce a retrograde signal in response to photoinhibitory light intensities. Signalling specificity achieved by chloroplast-sourced H_2_O_2_ for signal transduction may be dependent upon the oft-observed close association of a proportion of these organelles with the nucleus. In this review, we consider more precisely the nature of the close association between a chloroplast appressed to the nucleus and the requirement for H_2_O_2_ to cross both the double membranes of the chloroplast and nuclear envelopes. Of particular relevance is that the endoplasmic reticulum (ER) has close physical contact with chloroplasts and is contiguous with the nuclear envelope. Therefore, the perinuclear space, which transducing H_2_O_2_ molecules would have to cross, may have an oxidising environment the same as the ER lumen. Based on studies in animal cells, the ER lumen may be a significant source of H_2_O_2_ in plant cells arising from the oxidative folding of proteins. If this is the case, then there is potential for the ER lumen/perinuclear space to be an important location to modify chloroplast-to-nucleus H_2_O_2_ signal transduction and thereby introduce modulation of it by additional different environmental cues. These would include for example, heat stress and pathogen infection, which induce the unfolded protein response characterised by an increased H_2_O_2_ level in the ER lumen.

## 1. Introduction

Chloroplast-to-nucleus (retrograde) signalling is an important part of plants’ capacity to sense and act upon changes in their environment, especially those that require eventual adjustments to photosynthetic capacity. The ability to coordinate immediate and longer-term responses to environmental perturbations occurs at the cellular, tissue and whole plant (systemic) level [1,2,3,4,5,6,7]. A particularly active area within this research sphere is the quest to identify the precise signalling routes between chloroplasts and the nucleus. Several signalling pathways and signal initiators and transducers have been identified and continue to attract attention, although there are undoubtedly many more to be uncovered [8,9,10,11,12,13].

The close association of a proportion of a cell’s chloroplast complement with its nucleus is a feature of all plant species so far examined [11,14,15]. More recently, this relationship has received growing attention since the juxtaposition of a subset of chloroplasts with the nucleus is suggested to be a crucial feature in the communication and coordination of highly complex processes between these organelles in response to developmental and environmental cues. [4,11,16,17,18]. Since some signalling molecules could originate from multiple cellular sources, the close association between the nucleus and a subset of chloroplasts may provide the necessary specificity for retrograde signal transduction. Conversely, if no discrimination between the origins of such molecules was accommodated, then using them as signal transducers from the chloroplast would not provide any specificity [11,15]. The molecule where this argument is most pertinent and will be the example used in this essay, is hydrogen peroxide (H_2_O_2_) whose origin from different subcellular sources produces differential gene expression patterns, which implies that there is an associated signalling specificity [19,20,21,22].

In the context of retrograde signalling, specificity could be achieved by conversion of the oxidising equivalent from H_2_O_2_ to another molecule in the chloroplast [2,23,24,25]. While this does indeed occur, observations also suggest that H_2_O_2_ can also be the transducing signal from chloroplasts to the nucleus [4,16]. In higher plants, the movement of H_2_O_2_ between chloroplasts and the nucleus has been studied in *Nicotiana benthamiana* (*Nb*) epidermal pavement cells. This tissue is readily accessible for monitoring changes in the oxidation state of transiently expressed genetically encoded H_2_O_2_-reporting fluorescent biosensor proteins using confocal laser scanning microscopy [4,16,26]. Important for interpretation of responses to some environmental stresses is that *Nb* epidermal pavement cells are photosynthetic [4]. The H_2_O_2_ that accumulates in *Nb* chloroplasts in these studies arises in response to increased light intensity or to pathogen effector triggered immunity [4,16,27]. However, a wide range of environmental challenges cause changes in H_2_O_2_ levels in other subcellular compartments including the peroxisome, mitochondrion, cytosol and the plasma membrane [5,13,22,28,29,30]. Therefore, chloroplast-nucleus association is proposed to be relevant in determining how H_2_O_2_ secreted from chloroplasts [31] could be specific in the transduction of an oxidising signal to the nucleus [4,11,15].

The aim of this short article is not to provide a detailed consideration of all aspects of chloroplast-nucleus association but rather to consider the route H_2_O_2_ may take in its journey from the chloroplast to the nucleus. Despite the apparently short distance of travel between the origin and destination for H_2_O_2_ in retrograde signalling, we reflect here that other factors and subcellular environments could influence both the potency and specificity of this transducing signal.

## 2. Stromules

Effector triggered immunity in *Nb* pavement cells elicited by flagellin, chitin, INF1 (an extracellular *Phytophthora infestans* protein) or over-expression of NADH dehydrogenase-like (NDH) complex M subunit, causes chloroplast aggregation around nuclei and the formation of tubular chloroplast stroma extensions (stromules) [16,26,32,33]. Stromule formation may be associated with a suppression of photosynthesis, which occurs in *Arabidopsis thaliana* challenged with elicitors [27]. Photoinhibition may also be an important step, which stimulates stromule formation in the absence of pathogen infection such as in senescing leaves [15]. Stromules appear to promote chloroplast-to-chloroplast contacts but also that of chloroplasts-to-nucleus [26,34] and are suggested to be conduits for H_2_O_2_ and selected proteins to transfer to the nucleus [16] although this remains under debate [26,34]. Stromules may also facilitate the clustering of chloroplasts with the nucleus since they have been shown to move along microtubules and anchored by actin filaments [32]. In addition, pathogen-derived effectors may also achieve the same end without stromules by promoting peri-nuclear clustering of chloroplasts [17,35]. 

## 3. Nature of the Linkages—The Nuclear Envelope

**Figure 1:**
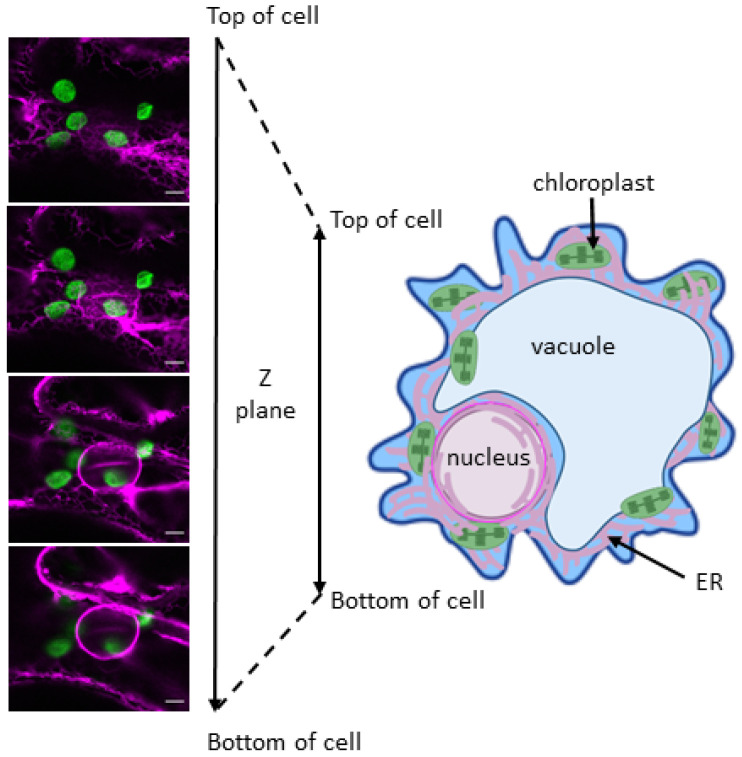
**The nuclear envelope, contiguous endoplasmic reticulum (ER) and chloroplasts are closely associated.** The four vertical panels on the left are selected top-to-bottom Z planes of a *Nicotiana benthamiana* abaxial epidermal cell transiently expressing the ER luminal marker RFP-HDEL (magenta) with chloroplast autofluorescence (green). The images were taken by confocal scanning laser microscopy. Scale bar, 5mm. The diagram on the right provides a pictorial interpretation of the combined Z planes. The thicker magenta circle is the nuclear envelope which is connected to the pink lines representing the ER. (Cell schematic created with BioRender).

The outer membrane of the nuclear envelope is continuous with the endoplasmic reticulum membrane (ER; Figure 1) [35] and consequently, the ca. 50 nm wide perinuclear space between the inner and outer nuclear membrane is contiguous with the ER lumen [36]. Chloroplasts, like many other organelles that form physical interactions with the ER, are tethered to the outer ER/nuclear membrane typically at 10–30 nm distance [37,38,39,40]. The ER outer membrane is thus frequently in very close association with the outer chloroplast envelope membrane [11,41,42,43]. The transient tethering of chloroplasts to the ER occurs at so-called membrane contact sites (MCS), which have been defined as “areas of close apposition between the membranes of two organelles” but crucially, the two organellar membranes do not fuse [38]. MCS are regarded as having specific functions, acting to concentrate protein-protein interactions to allow transfer of molecules between compartments [38]. The bidirectional exchange of lipids between the ER and chloroplasts via such MCS has been studied to some extent. Notably, transorganellar complementation experiments elegantly demonstrated the existence of metabolic continuity in biosynthetic pathways, which span both organelles [44,45]. These tethers between chloroplasts and the ER are such that a 400 pN force applied with optical tweezers could not separate them [39,46,47]. Various biophysical, genetic, biochemical and microscopy methodologies have begun to provide a picture of the complexity of these interactions and the reader is referred to the comprehensive review on this subject by Baillie et al. [39].

A long-observed phenomenon is the avoidance response of chloroplasts whereby they move away from high fluence blue light, which is controlled by phototropins and uses the actin cytoskeleton to guide movement [48,49]. Interestingly, the nucleus, which has no capacity to move independently, is towed by its attached chloroplasts [50]. Undoubtedly, many proteins are involved in the combined tethering of chloroplasts to nuclei and their repositioning in the cell, as well as being involved in other functions such as anchoring of plastids to the plasma membrane and chloroplast division. Examples include CHLOROPLAST UNUSUAL POSITIONING1 (CHUP1), KINESIN-LIKE PROTEIN FOR ACTIN-BASED CHLOROPLAST MOVEMENT1 (KAC1) and KAC2, PLASTID DIVISION1 (PDV1) and PDV2 and PARALOG OF ARC6 (PARC6) [15,50,51,52,53,54,55,56].

From a structure-function perspective, CHUP1 currently is one of the best-understood proteins engaged in chloroplast relocation and positioning [57]. CHUP1 localises to the chloroplast envelope and to do this requires the first 25 N-terminal residues, which form a hydrophobic domain. The remainder of the protein protrudes outwards into the cytosol. A coiled-coil region (residues 65–276), an F-actin binding region (residues 350–360) and proline-rich region (residues 670–710) ensure the anchoring of the chloroplast to the plasma membrane and linking it to the actin cytoskeleton and/or its polymerisation. Completing the protein is a conserved C-terminal region (residues 720–1004) which binds profilin [58]. CHUP1 forms homodimers via leucine zippers contained within its N-terminal coiled-coil region [59] and has the effect of bringing the proline-rich and actin binding domains into close proximity [59]. Most recently, it has been shown that the conserved C-terminal region also forms dimers and is a novel plant-specific actin nucleator sharing structural homology, but not sequence homology, to the FH2 C-terminal domain dimers of formins that regulate actin polymerisation across the *Eukarya* [60,61]. It should be emphasised that the aforementioned studies did not specifically address nuclear-chloroplast connectivity having focussed instead on chloroplast-plasma membrane connectivity. Nevertheless, one important observation is that CHUP1 may be a negative regulator of stromule formation [16] and in addition there is, to our knowledge, no information on how or even if CHUP1 is part of chloroplast-ER/outer nuclear membrane MCS. It was suggested recently that direct contact between plastids, the nucleus, and the same connections involving stromules are a continuum of essentially the same process and may provide a means of distinguishing the role of CHUP1 in chloroplast-nuclear connections from that of chloroplast-plasma membrane association [15].

One further consideration in this inter-organellar communication is the role of nuclear pore complexes (NPCs) [62,63,64] which punctuate the nuclear membranes. This is the route for the trafficking of macromolecules, most commonly proteins and nucleic acids [62,63]. This could be a route for the transfer of proteins engaged in retrograde signalling such as WHIRLY1 [65]. However, it is not clear that small molecules enter the nucleus via NPCs. Therefore, while it is a theoretical route for trafficking H_2_O_2_ or an oxidising equivalent as an oxidised protein there is no evidence of this and therefore no further consideration of NPCs will be undertaken here.

## 4. H_2_O_2_, Aquaporins and the Route to the Nucleus

From the above considerations, it can be proposed that there is close association between some of a cell’s complement of chloroplasts and the nucleus, which would also involve both organelles tied into the cytoskeleton with the strength of the connections determined by tethering through MCS. More precisely, for H_2_O_2_ to travel from the chloroplast stroma to the nucleus then it must not only cross the chloroplast double envelope, but also the outer and then inner nuclear membrane separated by the perinuclear space.

**Figure 2:**
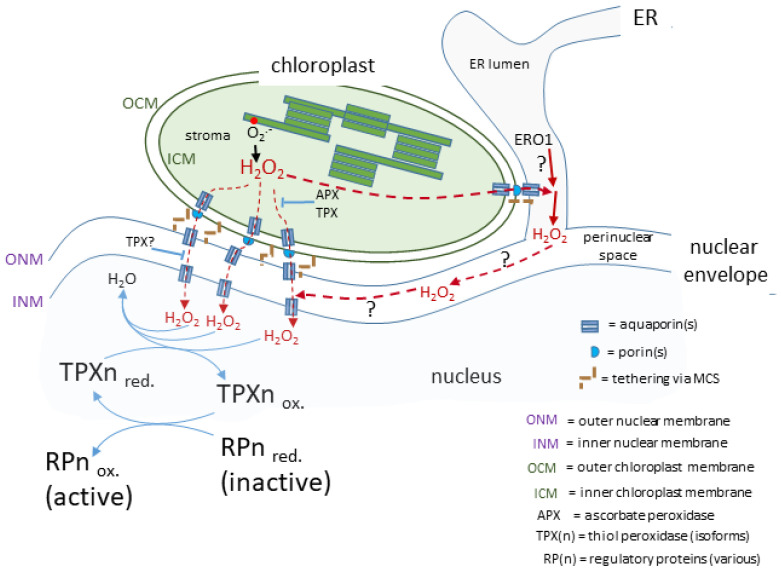
**A proposed route for a transducing H_2_O_2_ retrograde signal.** In this case, the chloroplasts and nucleus are in close association linked by the nuclear envelope and possibly influenced by H_2_O_2_ produced in the ER lumen. The H_2_O_2_ generated by photosynthetic electron transport passes through membranes facilitated by aquaporins and arrives in the nucleus to transfer its oxidising equivalents to a redox relay network ultimately leading to the activation of a range of diverse regulatory proteins, which may act in the nucleus or migrate to other subcellular sites.

The movement of H_2_O_2_ across membranes is considered to occur by diffusion down a concentration gradient facilitated by membrane intrinsic proteins (aquaporins; AQPs; reviewed by Bienert and Chaumont [66]). However, H_2_O_2_ diffusion into red blood cells is not facilitated by AQPs but by an unknown membrane protein or through the lipid fraction [67] raising the possibility of AQP-independent means of transporting H_2_O_2_ between cellular compartments. This is despite physico-chemical considerations concluding that simple diffusion of H_2_O_2_ across membranes can be disregarded [66,67]. Instead, all AQPs that transport H_2_O may also transport H_2_O_2_, although there are differences in the efficiency of how individual AQP isoforms discriminate between these two molecules [66,68,69]. Assuming a uni-directional movement of signal-transducing H_2_O_2_ to the nucleus from attached chloroplasts, then its journey would include crossing the chloroplast envelope membranes (Figure 2). Isolated chloroplasts exposed to high light intensities secrete H_2_O_2_ into their medium [31] and this is blocked by the AQP inhibitor acetazolamide [70]. Of the 35 AQPs in Arabidopsis [71], up to 5 may be present in the chloroplast. Of these, at least two isoforms of the tonoplast intrinsic protein (TIP1;1 and TIP1;2) AQP family and one of the plasma membrane intrinsic proteins, PIP2a, may span the inner chloroplast envelope membrane [72,73,74]. Therefore, the current evidence strongly suggests that AQPs are the exit route out of the chloroplast for H_2_O_2_.

The likelihood of very close contact between the chloroplast envelope and the outer nuclear envelope (see above) could include a localised increased concentration in microdomains at or near MCS and, if there is close proximity of further AQPs in the outer nuclear membrane, this would facilitate the transfer of H_2_O_2_ to the perinuclear space. Mitochondrial-ER MCS in animal cells form an environment where H_2_O_2_ does indeed concentrate in microdomains either side of the mitochondrial envelope [75]. It can be surmised that an analogous arrangement around chloroplast-outer nuclear/ER membrane could exist and certainly H_2_O_2_ microdomains have been observed associated with *Nb* epidermal chloroplasts [4]. Once in the perinuclear space, H_2_O_2_ would be in an oxidising environment (see following section) and therefore would have time to diffuse to the vicinity of any AQPs located on the inner nuclear membrane for its entry into the nucleus.

It should be emphasised that these considerations on the route from attached chloroplast to nucleus is informed speculation (Figure 2) based on the more complete information available from other eukaryotic cells. Whether this route for H_2_O_2_ actually exists in plant cells awaits experimental investigation. 

## 5. H_2_O_2_ in the Perinuclear Space and ER Lumen and Its Impact on Retrograde Signalling

In animal cells, the ER lumen is regarded, along with mitochondria and peroxisomes, as a major source of H_2_O_2_ for signalling [68,76,77,78]. These organelles are often found in very close proximity to each other and may secrete H_2_O_2_ into a shared microdomain in which proteins involved in further transducing the oxidising signal are also present. The cooperation between these three compartments to form a cytosol-located H_2_O_2_ microdomain has been termed the “redoxosome” [78]. A redoxosome for these same organelles but also including chloroplasts has been suggested as possible in plant cells, but this suggestion remains unexplored [79]. It has been proposed that in animal cells, the directing of H_2_O_2_ to the redoxosome ensures that it does not accumulate in the nucleus and cause oxidative damage there. However, plant cells subjected to environmental stress can accumulate chloroplast-sourced H_2_O_2_ in their nucleus [4,16]. This suggests that the organisation of the spatial components of H_2_O_2_-mediated retrograde signalling may differ from those involving non-plastid organelles, which may share a degree of conservation across the *Eukarya*.

**Figure 3:**
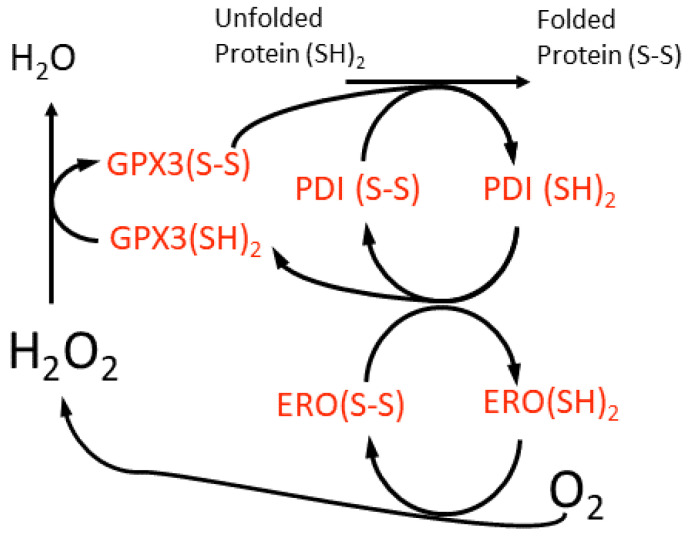
A scheme for the oxidative folding of proteins in the plant cell ER lumen and the generation of H_2_O_2_ by a luminal ER oxidase (ERO). This H_2_O_2_ may be scavenged by an ER glutathione peroxidase (GPX3), although the reductant for this enzyme is suggested to be protein disulfide isomerase (PDI) isoforms, which are members of the thioredoxin super-family. This proposed redox cycle is adapted from and available in more detail in the review by Meyer et al. [84].

The midpoint redox potential of the reduced glutathione-glutathione disulphide (GSH-GSSG) couple (*E*_GSH_) in the ER lumen is −208 ±4 mV, which is more oxidising than that of the cytosol at ca. −320 mV in animal cells [80]. However, very recent in vivo measurements conducted on Arabidopsis ER suggest a slightly more reducing *E*_GSH_ of −241 mV [81]. Irrespective of these differences between animal and plant cells, the ER lumen environment allows the chaperone-catalysed oxidative folding of proteins to occur that requires molecular oxygen (O_2_) and from which H_2_O_2_ arises (Figure 3). This is a highly conserved process in all eukaryotic cells. Oxidative stress in the ER is caused when this protein folding activity exceeds the capacity of the lumen antioxidant system to remove the H_2_O_2_ formed. GLUTATHIONE PEROXIDASE7 (GPX7), GPX8 and PEROXIREDOXIN4 (PRDX4) scavenge the H_2_O_2_ generated by the ER oxidoreductase1 (ERO1)-catalysed oxidation of the PROTEIN DISULPHIDE ISOMERASE (PDI) isoforms (Figure 3). Despite their names, GPX7 and GPX8 use reduced PDI isoforms as electron donors and not GSH [77,82]. There are also additional ERO1-independent means of generating H_2_O_2_ [83].

The increased H_2_O_2_ levels in the ER lumen can drive signalling, most notably the initiation of the Unfolded Protein Response (UPR), which acts to mitigate against the accumulation of unfolded or misfolded proteins in the ER lumen. One branch of the UPR is mediated by a pair of ER membrane-associated bZIP transcription factors—bZIP17 and bZIP28. UPR is also activated as a consequence of environmental perturbations including exposure to heat/chilling stress, oxidative stress, salt stress, induction of immunity and senescence [79,85,86,87].

## 6. Suppression of the UPR by High Light Intensities

The transfer of H_2_O_2_ from high light–exposed chloroplasts to their associated nucleus is an important step in the retrograde signalling mediated by this reactive oxygen species (ROS) [4,11,88]. Interestingly, exposure to high light suppresses the UPR, which is linked to the production of the ROS singlet oxygen (^1^O_2_) [87]. This is achieved by activation of the bZIP transcription factor LONG HYPOCOTYL5 (HY5), which competes with bZIP28 for binding to the promoters of UPR-activated genes and suppressing their induction [89]. The HY5-mediated negative regulation of the UPR involving ^1^O_2_ may be linked to the recent identification of HY5 as a positive regulator of high light acclimation [90]. This is because the relative levels of ^1^O_2_ and H_2_O_2_ may be a good indicator of the type of physiological response a plant carries out when exposed to increased light intensities [88,90,91,92,93].

## 7. Conclusions and Possibilities

The corollary of the above arguments is that during transit across the perinuclear space—an extension of the ER lumen—there could be the opportunity to modulate retrograde signalling mediated by H_2_O_2_ from the chloroplast on its way to the nucleus. One can envisage two converse scenarios: (a) increased H_2_O_2_ from the ER lumen augmenting H_2_O_2_ coming from chloroplasts and amplifying a stress-responsive signal; or the opposite: (b) the attenuation of a retrograde signal at this point by increased and highly localised antioxidant activity. These possibilities now have the prospect of being tested with the advent of a novel GSH:GSSG redox biosensor that functions in the plant ER lumen [79] together with the possibility of using a modified Hyper, called Triper, to detect H_2_O_2_, which elegantly sidesteps problems of this biosensor’s over-oxidation and its consequent non-responsiveness [77].

In conclusion, if the considerations in this essay are correct then this could provide a means of intervening in retrograde signalling to tailor a crop plant’s response to environmental stress [13]. This may prove to be an easier option than trying to manipulate a H_2_O_2_ signal once it has arrived in the nucleus considering the transfer of oxidising equivalents is likely through an extensive and highly mobile network of intermediate redox carriers [25,94,95] to a plethora of recipient redox sensitive regulatory proteins.
